# Evolution of the cAMP-dependent protein kinase (PKA) catalytic subunit isoforms

**DOI:** 10.1371/journal.pone.0181091

**Published:** 2017-07-25

**Authors:** Kristoffer Søberg, Line Victoria Moen, Bjørn Steen Skålhegg, Jon Kristen Laerdahl

**Affiliations:** 1 Department of Nutrition, Institute of Basic Medical Sciences, University of Oslo, Oslo, Norway; 2 Department of Molecular Medicine, Institute of Basic Medical Sciences, University of Oslo, Oslo, Norway; 3 Department of Microbiology, Oslo University Hospital Rikshospitalet, Oslo, Norway; 4 Bioinformatics Core Facility, Department of Informatics, University of Oslo, Oslo, Norway; Indian Institute of Science, INDIA

## Abstract

The 3’,5’-cyclic adenosine monophosphate (cAMP)-dependent protein kinase, or protein kinase A (PKA), pathway is one of the most versatile and best studied signaling pathways in eukaryotic cells. The two paralogous PKA catalytic subunits Cα and Cβ, encoded by the genes *PRKACA* and *PRKACB*, respectively, are among the best understood model kinases in signal transduction research. In this work, we explore and elucidate the evolution of the alternative 5’ exons and the splicing pattern giving rise to the numerous PKA catalytic subunit isoforms. In addition to the universally conserved Cα1/Cβ1 isoforms, we find kinase variants with short N-termini in all main vertebrate classes, including the sperm-specific Cα2 isoform found to be conserved in all mammals. We also describe, for the first time, a PKA Cα isoform with a long N-terminus, paralogous to the PKA Cβ2 N-terminus. An analysis of isoform-specific variation highlights residues and motifs that are likely to be of functional importance.

## Introduction

The protein kinase (PK) gene family is one of the largest in the human genome, comprising over 500 different PK encoding genes [1]. PKs catalyze the transfer of phosphate groups onto Ser, Thr, or Tyr residues of target proteins. Phosphorylation of substrates represents a key regulatory mechanism in all eukaryotic cells [1], and the various PKs target different substrates with a multitude of biological effects. 3’,5’-cyclic adenosine monophosphate (cAMP)-dependent protein kinase, or protein kinase A (PKA), is among the best studied PKs. It has been used as a model kinase for all PKs, and several thousand articles and over a hundred different crystal structures of PKA have been published, emphasizing its key role in PK and signal transduction research.

In humans, inactive PKA is composed of two regulatory (R) subunits forming a dimer, and two catalytic (C) subunits bound to the R subunits, together building the inactive tetrameric holoenzyme. Activation of PKA catalytic activity is initiated by any signal causing an increase in intracellular cAMP concentration. The traditional view of PKA activation is that two cAMP molecules bind to each R subunit, causing a conformational change in the R subunit dimer, and the C subunits are released and become catalytically active through exposure of their active sites [2]. It should be noted that there is increasing evidence that some C subunits may not fully dissociate from the R subunits upon cAMP stimulation, but rather phosphorylate their substrates in close proximity to the R subunits [3]. For normal functioning of PKA signaling, it is critical that PKA activity is tightly regulated in space and time. This is achieved at several levels, among these through the use of alternative R and C subunit isoforms. Many PKA holoenzymes are also typically anchored to subcellular structures via the R subunits, which may form docking motifs upon dimerization, and in turn may bind to scaffolding proteins known as A kinase anchoring proteins (AKAPs) [4]. C subunits may also be directed to specific subcellular locations through direct binding of the C subunits to interaction partners, as has been demonstrated in the case of AKIP1. AKIP1 docks to the N-terminus of the C subunit and promotes its retention in the nucleus [5, 6].

All PKs share the same overall catalytic core 3D structure that is necessary for catalytic activity. In PKA, this segment is encoded in the C subunit genes. Five different established or putative C subunit encoding genes have been identified in the human genome; *PRKACA*, *PRKACB*, *PRKX*, *PRKY*, and *PRKACG* [7–12]. The PKA C subunit encoded by *PRKACA* was the first PK for which the 3D structure was solved [13], and it has been extensively studied the last decades. Several structure-function-relationships encoded by *PRKACA* have proved to be conserved features of PKs in general [14–16]. The conserved catalytic core consists of an N-terminal lobe, called the small lobe or N-lobe, comprising mainly β-strands and a single α-helix. Located C-terminally is the large lobe or C-lobe, consisting mostly of α-helices. Between the small and large lobes is the active site cleft which binds an ATP molecule and two divalent cations, preferentially Mg^2+^ [17], whereas Ca^2+^ may also play an important physiological role [18]. These cations are essential in the process of transferring the γ-phosphate of ATP to Ser or Thr residues on PKA substrates. The N- and C-terminal parts of PKs outside of the conserved catalytic core are termed the N- and C-tail, respectively [19]. The N- and C-tails are more variable across the human kinome (*i*.*e*. the kinase-encoding part of the human genome), and may convey more subgroup- or isoform-specific functions of a PK. In the AGC subfamily of PKs, which includes PKA and the subgroup of PKs that have the highest degree of sequence identity to PKA, cGMP-dependent protein kinase (PKG), and protein kinase C (PKC) [1, 20, 21], the C-tail shares conserved features important for AGC kinase functioning, including a docking site for the AGC kinase activator PDK1 [21]. In contrast, the N-tail is not highly conserved among AGC kinases, and may therefore provide a more subtle regulation of subgroup-specific functions of these PKs [19]. As described below, the N-tail is highly variable even among the different PKA C subunits.

The best studied and major cAMP-dependent catalytic activity in human cells comes from the proteins Cα and Cβ encoded by *PRKACA* and *PRKACB*, respectively. We have previously shown that *PRKACA* and *PRKACB* are highly conserved paralogous genes as a result of a gene duplication event around the evolution of the first vertebrate species some 500 million years ago [22]. The main splice variants of the human proteins, termed Cα1 and Cβ1, share 93% sequence identity, and have the same length, 350 residues. In the same study we also identified eleven amino acid positions that uniquely define the Cα and Cβ protein clades. These sequence differences may be associated with functional differences and hence in part explain why they are both universally conserved in the bony vertebrates [22]. With the exception of brain-specific deletion mutants in *PRKACB* lacking exon 4 (CβΔ4) [23], all Cα and Cβ proteins share the same conserved core in the segment known as Core_16-350_, consisting of Cα1/Cβ1 residues 16 to 350. These residues are encoded by *PRKACA* and *PRKACB* paralogous exons 2 to 10 [22]. Core_16-350_ includes the entire catalytic core domain (Cα1/Cβ1 residues 40–300) and it is therefore likely that all respective Cα and Cβ splice variants, excluding CβΔ4, are kinetically similar, as has been experimentally demonstrated in the case of Cα [24]. This study also showed no difference in RI and RII subunit binding affinities for the two main Cα variants.

Our previous analysis did not consider variability in the DNA sequences encoding the N-termini of Cα and Cβ. Both *PRKACA* and *PRKACB* encode several protein variants due to alternative use and splicing of exons located 5’ to the conserved exon 2. Human *PRKACA* has two alternative 5’ exons, exon 1–1 and exon 1–2, giving rise to the proteins Cα1 and Cα2, respectively. Whereas Cα1 is ubiquitously expressed in human, Cα2 appears to be exclusively expressed in sperm cells [25–27]. Deletion of the *PRKACA* gene in mice leads to growth retardation and early postnatal lethality, as well as male infertility among the mice that do grow up [28, 29]. Whereas the human Cα1 N-terminus is encoded by the 14 residue sequence (M)GNAAAAKKGSEQES(V) (encoding of Val15 is shared with exon 2, intron phase 1), the N-terminus of human Cα2 is encoded by the six residue sequence (M)ASNSSD(V) (Val7 codon spanning intron 1). In Cα1 the N-terminal Met residue is co-translationally removed by methionine amino-peptidases [30], and this is likely the case also for Cα2 [27].

The function of several residues in the Cα1 N-terminus has been investigated. This segment is modified by several post-translational modifications (PTMs), including myristoylation of Gly1, deamidation of Asn2, as well as phosphorylation of Ser10 [31–35]. These PTMs have been demonstrated experimentally to have functional consequences for mammalian Cα1 subunit function and 3D structure conformation, as well as subcellular localization. Experiments have indicated that deamidation of Asn2 into Asp2 results in C subunits with a tendency to remain in the cytoplasm compared to the Asn2 form, reflected in reduced accumulation of C subunit in the nucleus and consequent reduced PKA-mediated phosphorylation of CREB [36]. Cα2, previously only studied and described in human [25, 26], mouse [25, 26], and ram [27, 37], has a sperm-specific expression pattern, and specific deletion of exon 1–2 of *PRKACA* in mice leads solely to male sterility [29] in otherwise healthy mice. It is currently not known if this splice variant is conserved in mammals or if it is found in other vertebrates. Compared to Cα1, much less is known about potential functions of specific residues in Cα2. The modifiable residues at the Cα1 N-terminus are missing in Cα2.

The human *PRKACB* gene has four alternative 5’ exons, exon 1–1, 1–2, 1–3, and 1–4, in addition to a cassette of short exons denoted a, b, and c, that are known to be alternatively combined with either exons 1–3 or 1–4. Alternative use of exons located 5’ to exon 2 in the *PRKACB* gene gives rise to at least 10 and potentially more than 16 different catalytically active Cβ proteins [8, 12, 38–41]. Human Cβ1 contains the same modifiable N-terminal residues as seen in Cα1, namely Gly1, Asn2, and Ser10, and the 5’ coding exon 1–1 in *PRKACB* is very likely a paralogous exon of *PRKACA* exon 1–1. Cβ1 is ubiquitously expressed [40], and is the only Cβ protein that has an N-terminal sequence compatible with modification by myristoylation [8]. *PRKACB* exon 1–2 encodes a long N-terminus of the Cβ2 protein, comprising 63 residues. Cβ2 has been described in several species, including human [40], cow [12], pig, rat, mouse, and turkey [42]. Little is known of the function of the Cβ2-specific N-terminal end, but it includes a stretch of residues predicted to form an amphipathic α-helix, opening for the possibility of membrane targeting [12]. In contrast to Cα1 and Cβ1, Cβ2 expression varies highly among different tissues, with the highest expression in lymphoid tissues [38, 40]. Exons 1–3 and 1–4 encode the neural-specific proteins Cβ3 and Cβ4, respectively. Exons a, b, and c are only seen in combination with exons 1–3 or 1–4, and are therefore also neural-specifically expressed [8, 23, 39, 40, 43].

Evolution of the N-terminal variants of the Cα/Cβ paralogs has not been examined before. We previously elucidated potentially functionally important differences between the Cα and Cβ Core_16-350_ segments. However, as a major part of heterogeneity in the extremely conserved *PRKACA* and *PRKACB* genes and their corresponding proteins are located in the diverse N-termini, it is likely that the significant differences in isoform-specific functions can be a result of alternative use of exons located 5’ of exon 2 in both *PRKACA* and *PRKACB* genes. We show that exon 1–1 is universally conserved in vertebrate PKA Cα/Cβ, and that the presence of short alternative 5’ exons also appears to be conserved in both paralogs. For the first time, a long 5’ exon in *PRKACA*, paralogous to *PRKACB* exon 1–2, is described, showing that several alternative PKA C transcripts in early vertebrates were duplicated as a result of the PKA C gene duplication. Our study highlights specific residues and motifs encoded by 5’ exons in *PRKACA* and *PRKACB* that are conserved to varying degrees throughout metazoan evolution, and gives clues to structure and function of specific PKA C subunit isoforms. Multiple sequence alignments of orthologous *PRKACA* and *PRKACB* alternative 5’ exons revealed highly conserved exons, residues and motifs that indicate functional importance.

## Results and discussion

### The PKA catalytic subunit gene was duplicated in a common ancestor of all Gnathostomata

We have previously shown that while the genomes of invertebrate animals, including the Tunicata and Cephalochordata (lancelets), encode a single PKA C subunit gene, *PRKACA* and *PRKACB* are paralogous genes that arose due to a gene duplication event in early vertebrates. In this previous study we curated and published a dataset of 81 full length and 15 partial PKA Cα/Cβ sequences [22]. The amount of publicly available genomic and transcriptomic sequences has increased dramatically since our previous study, and more data is now available also for the most early-branching lineages of vertebrates, the jawless and cartilaginous fishes. Full length sequences of the PKA Cα/Cβ homologs from Australian ghostshark (*Callorhinchus milii*) and sea lamprey (*Petromyzon marinus*) were collected and assembled and are listed in Supplementary S1 and S2 Tables.

Bayesian inference of phylogeny was carried out as described previously [22], with the same data, but now also including the sequences from early-branching vertebrates. Inclusion of the new data in the phylogeny calculations shows that cartilaginous fish indeed have both *PRKACA* and *PRKACB* and that the gene duplication leading to the two paralogs occurred in a common ancestor of Gnathostomata, the jawed vertebrates. The lamprey also has two PKA C subunit genes, and the most parsimonious explanation would be that the gene duplication occurred in a common ancestor of all vertebrates, including the jawless fishes. However, our phylogenetic investigations, despite employing state-of-the-art methods [22], were unable to confirm this and consequently also to classify the two lamprey paralogs as either PKA Cα or Cβ. This is not surprising given that the gene duplication occurred close to the splitting between jawed and jawless vertebrates, and that lamprey is known to have a strong amino acid and codon usage bias, resulting in problematic nonphylogenetic signals in phylogenetic studies [44]. The two lamprey PKA C subunit proteins are denoted PKA C homolog 1 and 2 below, and we believe the classification of either one as PKA Cα or Cβ will be close to impossible due to the very limited phylogenetic signals remaining in the relevant parts of the vertebrate genomes.

In our previous report on the PKA C subunit gene family, four years ago, we found no evidence of *PRKACA* orthologs in any bird or reptile species in published data, except for a single chicken (*Gallus gallus*) EST (expressed sequence tag). We now readily find *PRKACA* sequences from a number of reptile species, for example in Carolina anole lizard (*Anolis carolinensis*) (RefSeq protein identifier XP_016846888.1) and American alligator (*Alligator mississippiensis*) (XP_006271168.1). There are still no full-length PKA Cα sequences available in the sequence databases for birds, but thousands of transcriptome SRA reads from many species show that *PRKACA* is present in many, most likely all, bird genomes. We combined genomic and transcriptomic data and generated a full length PKA Cα1 sequence for the golden eagle (*Aquila chrysaetos*) (See S1 and S2 Tables). The *PRKACA* gene was completely missing from the chicken reference genome until early 2016, and we speculate that the gene is missing from nearly all other published bird genomes due to genome assembly at least partially guided by an incomplete chicken genome.

**Fig 1:**
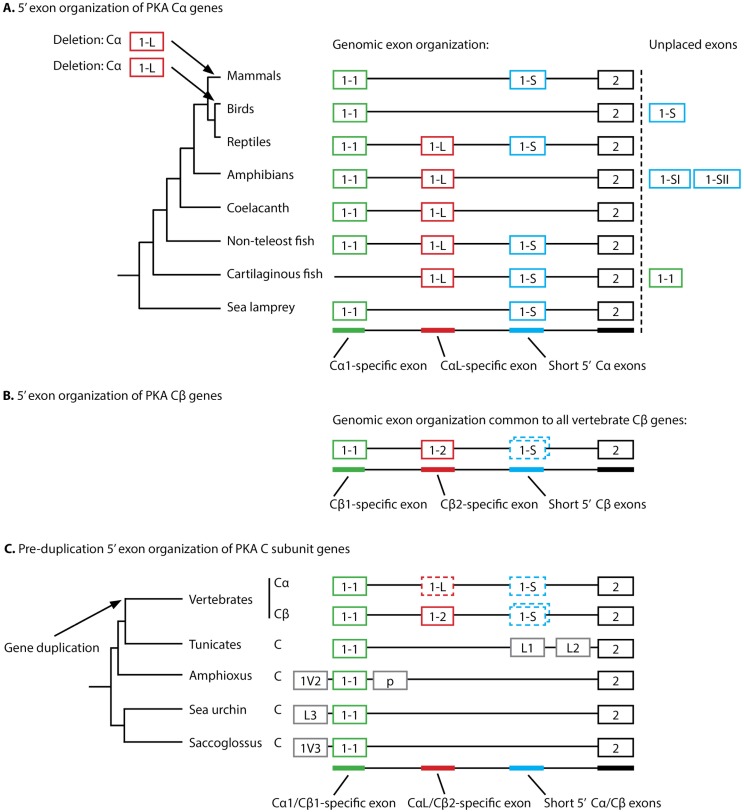
Phylogeny and organization of 5’ exons in *PRKACA/PRKACB* homologs. (A) The branching diagram (left) shows the major vertebrate classes or representative species from major classes and their evolutionary relationship. The organization of the different 5’ exons in *PRKACA* for each branch was determined based on data from genomic and transcriptomic sequence databases. Exons 1–1 (green), 1-L (red), and 1-S (blue) are represented as boxes and reflect the genomic ordering of 5’ exons encoding the Cα1, CαL and short Cα transcript variants, respectively. Exon 2 (black) is conserved in all species [22]. Exons that could not be placed due to missing genomic sequence coverage are also shown (Unplaced exons). In mammals, 1-S is identical to exon 1–2 encoding the Cα2 sperm-specific variant, and is conserved in all major mammalian groups. It is not possible to determine whether or not mammalian exon 1–2 is orthologous to the short 1-S exons in non-mammalian classes. CαL-specific sequences were not identified in mammals and birds, the most parsimonious explanation being two separate deletion events in these groups, as indicated by arrows. (B) For all major vertebrate groups shown in panel A, the organization of 5’ exons 1–1 (green) and 1–2 (red) in *PRKACB* are identical. In addition, several short exons 1-S (blue, dashed boxes) were identified in *PRKACB* between exon 1–2 and exon 2 (black). (C) The duplication of an ancestor C subunit gene occurred around the evolution of the first vertebrate species (arrow). 1-L and 1-S specific *PRKACA* exons and 1-S specific *PRKACB* exons not identified in all vertebrate groups are shown as dashed boxes. The exons p, 1V2, 1V3, L1, L2, and L3 denote various 5’ exons found in invertebrate species only. The tree shows tunicates as the closest living relatives of vertebrates [46], and the echinoderm sea urchin and hemichordate acorn worm (*Saccoglossus kowalevskii*) as belonging to sister phyla [47].

**Fig 2:**
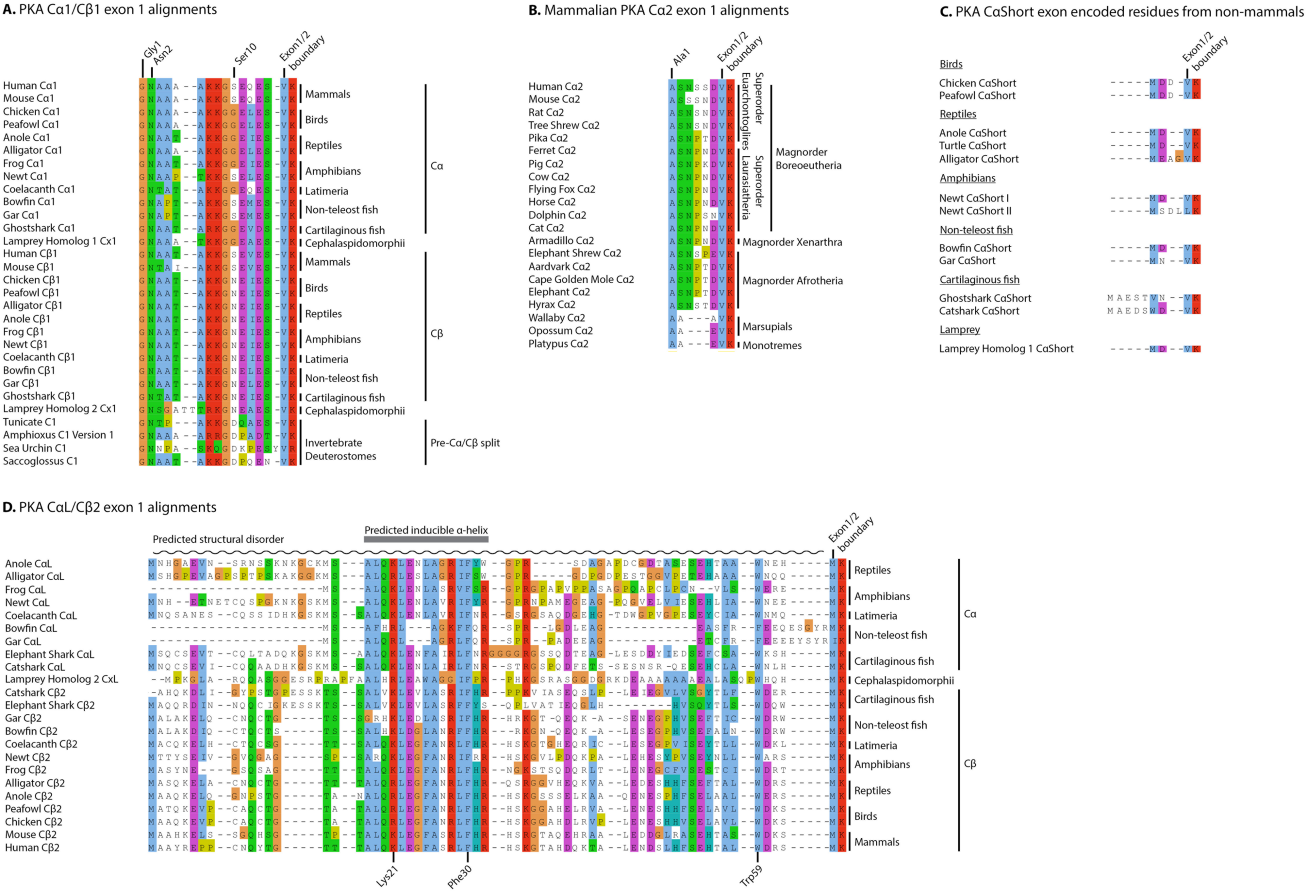
Multiple sequence alignments of PKA Cα/Cβ N-termini. (A) Alignment of the segments encoded by Cα1- and Cβ1-specific 5’ exons from selected deuterostomes, including *Homo sapiens*, *Mus musculus*, *Gallus gallus*, *Pavo cristatus*, *Anolis carolinensis*, *Alligator sinensi*s, *Xenopus tropicalis*, *Cynops pyrrhogaster*, *Latimeria chalumnae*, *Amia calva*, *Lepisosteus oculatus*, *Callorhinchus milii*, *Petromyzon marinus*, *Ciona intestinalis*, *Branchiostoma floridae*, *Strongylocentrotus purpuratus*, and *Saccoglossus kowalevskii*. The N-terminal Met residue is removed *in vivo*, and the alignment starts at Gly1. The alignment also includes the transition to exon 2, Val15 in human PKA Cα1/Cβ1 being encoded by both exon 1 and exon 2 (intron phase 1), while Lys16 is encoded by exon 2. (B) Alignment of residues encoded by Cα2-specific exon 1–2 from all major mammalian groups. The N-terminal Met is most likely removed *in vivo* [27], and the alignment starts at Ala1. (C) CαShort sequences encoded by exons 1-S from published transcriptomes of selected non-mammalian species. Note that all sequences not necessarily are true orthologs with a common ancestor. The species are the same as listed in panel A, in addition to the catshark *Scyliorhinus canicula*. The N-terminal Met residue is included in the alignment. (D) Alignment of residues encoded by CαL- and Cβ2-specific 5’ exons from some of the species listed in panels A and C. The N-terminal Met residue is included in the alignment. All alignments are given with the Clustal X color scheme [48].

The nine paralogous 3’ exons, exons 2 to 10, of vertebrate *PRKACA* and *PRKACB* genes are expressed and translated in all known catalytically active protein isoforms. In addition, the genes have several exons located 5’ of exon 2 that are alternatively used/spliced with exons 2 to 10, but the evolution of these 5’ exons has not previously been thoroughly investigated. We performed extensive sequence searches in databases containing transcriptomic data, in particular the RNA-Seq data available from the NCBI SRA resource [45], for a large number of vertebrate species. The main focus was on investigating exon use and splicing 5’ of exon 2 in *PRKACA* and *PRKACB* (See S1 Methods and S1 Fig). Data from all major vertebrate groups was obtained, as well as from some few invertebrates. The genomic sequences were collected from genomic sequence databases of the same species, when these were available. The corresponding translated protein sequences for the *PRKACA* and *PRKACB* transcripts were aligned and analyzed. The evolution of 5’ exons in *PRKACA* and *PRKACB* is depicted in Fig 1, while multiple sequence alignments of the N-termini of the corresponding Cα and Cβ proteins are shown in Fig 2.

### *PRKACA* exon 1–1 and *PRKACB* exon 1–1 are paralogous exons

Transcriptomic data, mainly RNA-Seq raw sequence data, was collected, aligned, and carefully investigated and classified. We identified exons that were orthologs of human *PRKACA* exon 1–1 in all major vertebrate groups analyzed, including mammals, birds, reptiles, amphibians, the *Latimeria* coelacanth, teleost and non-teleost fish, cartilaginous fish, as well as lamprey, and denoted these exons 1–1 (Fig 1A, green boxes). Some of the corresponding protein sequences are aligned in Fig 2A. We have previously shown that additional gene duplications in teleost fish, giving rise to two *PRKACA* paralogs and two *PRKACB* paralogs [22], resulted in a particularly complicated PKA C gene family in teleost fishes. We identified several exons homologous to exon 1–1 in this group as well (data not shown), but teleost fish PKA C genes were not investigated further.

The genomic location of the exons 1–1 relative to other *PRKACA* exons were determined through genomic database searches of the respective species. It should be noted that in cartilaginous fish the genomic sequence coverage was incomplete, and exon 1–1 was only recognized in transcriptomic data, making it impossible to determine the exon organization in this group (Fig 1A, “unplaced exons”). Nevertheless, we have convincing evidence that exon 1–1 of *PRKACA* is universally conserved in vertebrates.

As for *PRKACA*, *PRKACB* transcripts including exon 1–1 were collected and analyzed. *PRKACB* exon 1–1 was found to be conserved in all classes of vertebrates (Fig 1B, green box). The N-termini of the corresponding Cβ1 protein sequences are shown aligned with PKA Cα1 in Fig 2A and strongly support *PRKACA* exon 1–1 and *PRKACB* exon 1–1 as paralogous exons.

### *PRKACA* has a long 5’ exon paralogous to *PRKACB* exon 1–2

Through our investigations on *PRKACA* exons located 5’ of exon 2 in vertebrate transcripts, we identified a novel, long 5’ exon between exons 1–1 and 2 conserved in most vertebrate classes (Fig 1A, red boxes, and Fig 2D). We denoted this previously not described *PRKACA* encoded “long” 5’ exon as exon 1-L. Cα encoded with this alternative 5’ exon will be referred to as CαL throughout this report. More than 50 bird and mammalian transcriptomes from a variety of tissues, as well as genomes, were analyzed using BLAST sequence searching for homologs of exon 1-L with no positive results, indicating that exon 1-L is missing from extant bird and mammalian genomes. The most parsimonious explanation for this finding is that exon 1-L was found in the ancestral *PRKACA* of all vertebrates, but that two separate deletion events have deleted the exon, and corresponding CαL transcript, in mammals and birds (Fig 1A, arrows, left).

In contrast to exon 1-L, the long exon 1–2 of *PRKACB* was identified in all major vertebrate groups (Fig 1B, red box, and Fig 2D), including mammals, as previously shown [12]. The alignment of the protein sequences corresponding to *PRKACA* exon 1-L and *PRKACB* exon 1–2 (Fig 2D) shows significant sequence similarity and strongly suggests that these two exons, as exons 1–1 and 2 to 10, are paralogous (Fig 1, red boxes “1-L” and “1–2”), and that they have not arisen independently.

### Evolution of vertebrate PKA C subunit short 5’ exons is non-trivial

Through our search of exons located 5’ of exon 2 in *PRKACA*, we detected short alternative 5’ exons in the transcriptomes of all major vertebrate groups except for the coelacanth (Fig 1A, blue boxes), the latter possibly due to the sparsity of sequence data for this vertebrate clade. We denoted these 5’ “short exons” as exon 1-S, and to be classified as short exons, they should not be longer than human *PRKACA* exon 1–2 encoding the Cα2 variant. Human *PRKACA* exon 1–2 encodes eight codons, including the start codon and the last codon that is split between exons 1–2 and 2. C subunits encoded with 5’ exon 1-S will be referred to as CαShort throughout this report. Selected sequences encoded by exon 1-S of various vertebrate CαShort proteins are summarized in Fig 2B and 2C. Genomic and transcriptomic sequences were compared, and showed identical exon organizations in all vertebrate genomes where genomic data was available, that is, exon 1-S is located between exons 1–1 and 2 or between exons 1-L and 2 when exon 1-L is present (Fig 1A). This is the same exon order as in all investigated *PRKACB* genomes, with short alternative 5’ exons located between exons 1–2 and 2 (Fig 1B). It should be noted that genomic sequence for the relevant *PRKACA* regions were lacking for birds and amphibians (Fig 1A), and we therefore denoted these exons “unplaced exons” in these species.

We identified exon 1-S in all main mammalian clades, including in marsupials and monotremes, as shown in the sequence alignment of mammalian sequences encoded by exon 1-S. (Fig 2B). Alignment of genomic sequences comprising exon 1-S and the flanking non-protein-coding sequences (*i*.*e*. CαShort 5’ UTR and intron 1 sequence) from major placental mammalian groups and the marsupial opossum, showed significant similarity for both noncoding and exon 1-S sequences (data not shown). Moreover, the sequence encoded by exon 1-S of the most basal mammal, the monotreme platypus (currently with poor genomic sequence coverage in relevant segment of *PRKACA*), was identical to opossum exon 1-S (Fig 2B). These findings strongly suggest that exon 1-S of all mammals, including marsupials and monotremes, are orthologs of human exon 1–2 and encode Cα2 proteins.

We were unable to determine whether exon 1-S from non-mammalian vertebrates (Fig 2C) are true orthologs of mammalian exon 1–2 (Fig 2B) or if these short exons have arisen independently multiple times. This is due to the exons being very short and not particularly conserved. In addition, it was not possible to reliably align the flanking non-protein-coding sequences (5’ UTR and intronic sequence), due to the long time span since the common ancestor of mammals and other vertebrates. Similarly, the weak phylogenetic signals that might have elucidated the evolutionary history of 1-S exons in *PRKACB* and their putative relationship with 1-S exons in *PRKACA* has been lost since the duplication of these genes.

We also investigated the expression pattern of mammalian exon 1–2, and found the Cα2 splice variant to be exclusively expressed in testis in all mammalian species analyzed, as previously found in mouse [25] and human [26]. The testis-specific expression pattern of exon 1-S was not found in any non-mammalian vertebrates. This may reflect different ancestry of non-mammalian exon 1-S, or may be evidence of an acquired testis-specific function of exon 1-S exclusively occurring in mammals. Based on this, we propose that the crucial function of Cα2 for male fertility is a shared trait of all mammals.

Finally, in the case of amphibians, two separate short 5’ exons were identified in several transcriptomes. The genomic location of these was not possible to determine, again, due to lack of genomic sequence data (Fig 1A, “unplaced exons”).

### 5’ exon-intron structure of *PRKACA/PRKACB* clarifies PKA C subunit evolution

We previously showed that the exon-intron structure of *PRKACA* and *PRKACB* exons 2 to 10 is conserved throughout vertebrate evolution [22]. Now, we show conservation also for the alternative *PRKACA* exons located 5’ of exon 2, and their paralogs in *PRKACB* (Fig 1A and 1B). This suggests the following set of events; 1) The pre-vertebrate PKA C subunit gene contained both an ancient 5’ exon 1–1 and an ancient long 5’ exon 1-L/1-2. 2) These two exons later developed into *PRKACA* exons 1–1 and 1-L, and *PRKACB* exons 1–1 and 1–2, respectively. It is unfortunate that the novel *PRKACA* exon 1-L and the corresponding transcript/protein CαL cannot be termed exon 1–2 and Cα2, respectively, as these labels are already well established in the literature as mammalian *PRKACA* short 5’ exon and corresponding testis-specific protein.

As described above, the evolutionary history of *PRKACA* exons 1-S is impossible to trace in non-mammalian vertebrates. However, given the conservation of exon-intron structure in all *PRKACA* genes investigated, as well as the location of the short exons 1–3 and 1–4 of *PRKACB* in mammals, it is possible that *PRKACA* exons 1-S are paralogous to one or more of the short *PRKACB* exons. Clarifying this is highly likely not possible due to the lack of phylogenetically informative sites of such short sequences, and was not attempted any further.

During our investigations of alternative transcription start and 5’ splicing of vertebrate *PRKACA* and *PRKACB* transcripts we did not find any reliable data supporting splice variants in addition to the ones already described above. In particular, no evidence for additional long or medium length 5’ exons were found, neither between exons 1–1 and 2 nor 5’ of exon 1–1.

### Heterogeneity in 5’ exons of pre-duplication PKA C subunit genes

In line with our hypothesis of *PRKACA* and *PRKACB* exons 1–1 being paralogs, we found similar exons in PKA C subunit genes of species that branched off from vertebrates prior to the *PRKACA* and *PRKACB* split (Figs 1C and 2A). In the same species, we found no evidence for exons homologous to *PRKACA*/*PRKACB* exons 1-L/1-2 (Fig 1C, red boxes). This may be due to insufficient sequence coverage, but more likely the exon has been deleted in these invertebrate species (as in mammalian and bird *PRKACA*) or it evolved in the common ancestor of vertebrates after the split with the invertebrates. Transcriptomic and genomic sequences showed highly diverse sequences in exons located 5’ of exon 2 in invertebrate species. We termed some of these exons L1, L2, L3, 1V2, 1V3, and p, where the latter was only found alternatively spliced in combination with exon 1–1 and three of the new exons were located 5’ of exon 1–1 (Fig 1C). The large variation in PKA C subunit 5’ splicing in invertebrates was not investigated further.

### Highly conserved sequences at the PKA Cα1/Cβ1 N-termini

**Fig 3:**
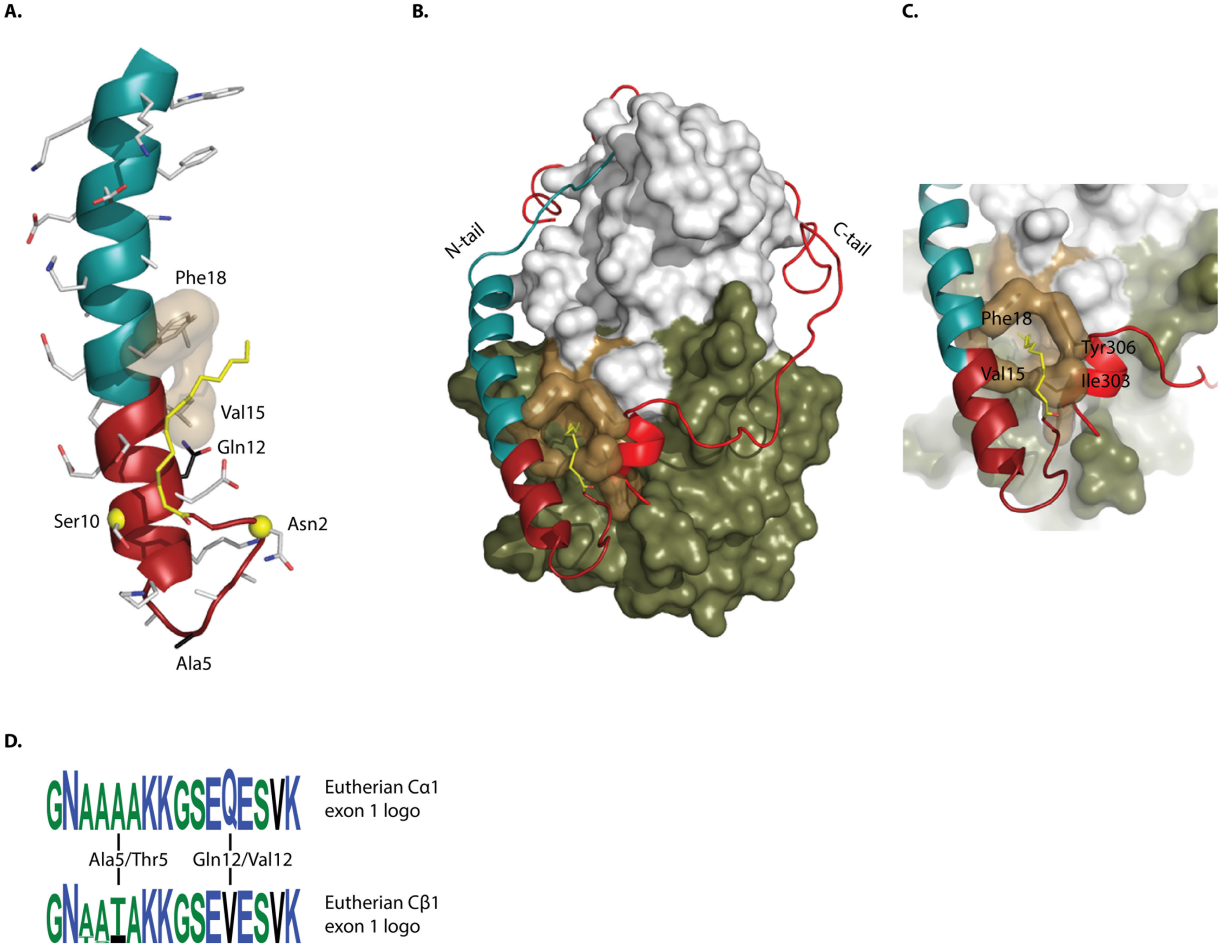
Structural model and sequence logo of the PKA Cα1/Cβ1 N-terminus. (A) Model of the N-terminal A helix of human Cα1. Residues encoded by exon 2 are presented in cyan, demonstrating that the A helix is elongated by the exon 1–1 encoded residues of Cα1 (dark red). The modifiable residues Asn2 and Ser10 are highlighted (yellow spheres on backbone Cα carbon atoms), and Gly1 is myristoylated (yellow). Val15 and Phe18 form part of the hydrophobic pocket. The eutherian Cα1-specific residues Ala5 and Gln12 are also highlighted. (B) Representation of the Cα1 N-terminus in relation to the full C subunit structure. Both the N-tail (cyan, except for exon 1–1 encoded part in dark red) and C-tail (red) form interactions with both the small (grey) and large (green) lobes. At their respective ends, they meet to form the entrance to the hydrophobic pocket (brown), composed of N-tail residues Val15 and Phe18, and C-tail residues Ile303 and Tyr306. The hydrophobic pocket may bind the myristic acid (yellow) in myristoylated C subunits. (C) Close-up of the entrance to the hydrophobic pocket, composed of residues Val15, Phe18, Ile303, and Tyr306. The model is based on the experimental structure by Zheng *et al*. [50] (PDB identifier 1CMK) (D) Sequence logos of PKA Cα1 and Cβ1 exon 1 encoded sequence from selected eutherian homologs (See S2 Fig). Lys 16 encoded by exon 2 is also included. The two paralogs are highly similar, with the notable exception of residues at positions 5 and 12, as indicated.

All published PKA C subunit 3D structures are of the Cα1 or Cα2 form, with varying degrees of structural order for the N-terminal residues [13, 35, 49, 50]. The structure reported by Zheng *et al*. [50] shows a myristoylated Cα1 subunit with a fully ordered N-terminus and the myristic acid docking into the hydrophobic pocket, as illustrated in Fig 3. All residues of the kinase domain that are interacting with the PKA Cα1 N-terminus or the attached myristic acid group in the hydrophobic pocket are absolutely conserved and identical in PKA Cα and Cβ in vertebrates (data from [22]). Several reports have indicated that myristoylated PKA C subunits may be regulated into “myr-in” and “myr-out” conformations with the myristic acid protruding either into the hydrophobic pocket of the C subunit (Fig 3), or away from the C subunit promoting membrane association [32–34]. The binding of C subunits to different R subunit isoforms is one way of regulating myr-conformation; myristic acid becomes solvent exposed and may bind to membranes upon C subunit binding to RII, but not RI subunits [33]. In addition to the myristoylation site at Gly1, two modifiable residues of the PKA Cα1 N-terminus have been described, Asn2 and Ser10, which may also provide means for regulating the myristic acid and Cα1 N-terminus conformation (Fig 3A) [31–34]. Asn2 may be deamidated and Ser10 may be phosphorylated, both increasing the negative charge of the N-terminus. The effects of these modifications on myr-conformation and membrane-affinity or other macromolecular interactions are not fully understood. The relation of the N- and C-tails to the rest of the myristoylated C subunit is depicted in Fig 3B, demonstrating that the two tails meet at their ends to form the entrance to the hydrophobic pocket (Fig 3C).

The high degree of conservation seen in the Core_16-350_ [22] of the Cα and Cβ proteins is also observed in the N-terminal 15 residues of Cα1 and Cβ1 (Fig 2A). With the exception of the lamprey homolog 2 Cx1 sequence, all sequences are of the same length. The Gly1 myristoylation and Asn2 deamidation sites are universally conserved in both Cα1, Cβ1, as well as in the C1 homologs from invertebrate species. This finding suggests that modification by myristoylation is a feature found in all vertebrate Cα1/Cβ1 subunits, and possibly all or most metazoan C1 subunits (Fig 2A). Ser10, however, was not found to be highly conserved among vertebrate Cα1/Cβ1 homologs. This residue is only conserved in both Cα1 and Cβ1 in mammals (Fig 2A). A more in depth analysis of different eutherian Cα1 and Cβ1 sequences from a range of species revealed that Ser10 was indeed conserved in all eutherian sequences investigated (S2 Fig). This may be a case of convergent evolution for this residue in Cα1 and Cβ1 proteins in mammals, and Ser10 appears to be functionally important in Cα1/Cβ1 in mammals, but not in other vertebrates. We note that all invertebrate C1 homologs investigated have negatively charged Asp encoded at this position.

N-myristoylation of proteins is usually not sufficient for membrane anchoring of proteins; they also require a second signal. This may, among other mechanisms, be achieved through basic amino acids forming electrostatic interactions with acidic phospholipids in membranes [30]. In myristoylated human PKA Cα1, Lys7 and Lys8 may serve this function. The presence of basic residues at position 7 and 8 is also a conserved feature of all vertebrate Cα1/Cβ1 homologs (Fig 2A).

Although the exon 1–1 encoded sequences of the Cα1/Cβ1 homologs are strikingly similar, there are also certain residues that do differ. In order to highlight differences in the mammalian Cα1/Cβ1 sequences, we created signature logos of the respective exon 1–1 encoded sequences from a selection of eutherian mammals (Fig 3D and S2 Fig). Two positions are consistently different in the Cα1 vs Cβ1 N-terminus, position 5 is invariably Ala5 in Cα1 and Thr5 or Ile5 in Cβ1, whereas residue 12 is invariably Gln12 in Cα1 and Val12 in Cβ1. Whether these variations have functional effects or not, such as affecting N-terminal stability and/or protein interactions, remains to be determined. It should also be noted that these residues are not conserved in non-mammalian groups, indicating that if they serve a conserved function, this is present only in mammals.

### PKA CαL and Cβ2 encode a long N-terminus with a conserved putative α-helix

The alignment of the CαL/Cβ2 N-termini revealed several conserved features. The segment previously predicted to encode an amphipathic α-helix in human Cβ2 [12] was the most conserved segment, and was identified in all CαL/Cβ2 homologs (Fig 2D, “predicted inducible α-helix”).

Approximately one third of all eukaryotic proteins contain intrinsically disordered regions (IDRs) spanning over 30 residues or more [51]. IDRs may act as flexible linkers connecting functional domains in proteins, but they may also contain regions that transiently fold upon interacting with other macromolecules. We collected full-length CαL and Cβ2 sequences (*i*.*e*. protein encoded by *PRKACA*/*PRKACB* exon 1-L/1-2 to exon 10) from selected vertebrates, including the coelacanth and Australian ghostshark CαL and Cβ2, as well as human Cβ2 and lamprey homolog 2 CxL. Without exception, structural disorder predictions for these sequences indicated the N-terminal segments to be IDRs (Fig 2D, “Predicted structural disorder”), whereas the remaining sequences encoded by exons 2 to 10 were predicted to be structurally ordered, as expected. Moreover, the majority of the disordered N-terminal sequences were predicted by DISOPRED3 [52] to contain a protein binding segment in the region encoding the putative inducible α-helix (Fig 2D, “Predicted inducible α-helix”). We propose that the CαL and Cβ2 isoforms both contain a disordered N-terminus, including a more conserved segment which in the presence of a yet unidentified interaction partner or under certain conditions may become ordered and protein binding, possibly in the form of an amphipathic helix (Fig 2D, “Predicted inducible α-helix”). The predicted IDR between the putative α-helix and the start of Core_16-350_ may function as a flexible linker.

The CαL/Cβ2 homologs do not contain an N-terminal Gly, hence they are unlikely to be myristoylated as in the case of Cα1 and Cβ1. This means that the hydrophobic pocket is either empty, or it is occupied by structures other than myristic acid from the N-terminus. Our multiple sequence alignment of CαL/Cβ2 homologs shows, with the exception of non-teleost fish, a universally conserved Trp in position 59 (human Cβ2 numbering) (Fig 2D). After analyzing previously published crystal structures of PKA Cα, it is tempting to propose a possible explanation for the conservation of this residue. Trp59 is located in reasonable distance in order for it to, based on its side chain hydrophobic properties, occupy the entrance to the hydrophobic pocket. This hypothesis needs to be experimentally verified. Positions 21 and 30 (human Cβ2 numbering) are always one of the two positively charged residues Lys or Arg, and universally Phe, respectively. Both of these residues are located in the putative inducible α-helix, and serve a conserved function which is yet to be determined. In contrast to the Cα1/Cβ1 N-termini, the length of the various CαL/Cβ2 N-termini varies significantly, and the sequence conservation in the segments outside of the putative α-helix is poor. Sequence conservation for the CαL/Cβ2 N-termini is particularly weak compared with the Core_16-350_ segment [22].

### PKA CαShort variants lack hydrophobic pocket-occupying residues or substituents

The crystal structure of Cα2 has been published [49]. This, together with NMR studies of Cα2 [49], have shown that purified Cα2 is able to bind aliphatic compounds resembling myristic acid. Based on these findings, it has been proposed that Cα2 may interact with Cα2-specific interaction partners binding to the unoccupied hydrophobic pocket [49]. Our alignment of eutherian Cα2 orthologs supports this hypothesis (Fig 2B). All collected Cα2 orthologs are short and lack a myristoylation site, in line with an unoccupied hydrophobic pocket. The alignment also shows a low degree of conservation of specific residues among the mammalian species. This is especially true when including the non-eutherian mammalian Cα2 sequences in the alignment, which we verified are indeed true orthologs of eutherian Cα2. The marsupial and monotreme Cα2 sequences are even shorter at the N-terminus than eutherian Cα2, and in this respect have more similarities to the non-mammalian CαShort and short Cβ variants [39], although the evolutionary relationship of these sequences is uncertain, most likely impossible to determine. We propose that the defining characteristic of Cα2 (Fig 2B), and CαShort in general (Fig 2C), is the lack of hydrophobic pocket-occupying substituents or residues.

### Future perspectives

We have in the current and previous [22] article elucidated the evolution of the genes encoding the main PKA C subunits Cα and Cβ in vertebrates. We have identified several distinguishing features of the various C subunits and their splice variants, and propose that these differences have functional consequences. An increasing amount of evidence suggests that different PKA C isoforms serve non-redundant functions [53–55], and we postulate that our studies can help explaining how the various C subunit variants may be implicated in specific biological processes and diseases. Since no 3D structures apart from Cα1 and Cα2 have been solved, it would be of high interest to determine the structure of additional C subunit isoforms. In particular, an experimental investigation of the structure of the long N-terminus of Cβ2 with its conserved, putative α-helix-encoding segment might elucidate features that can be associated with resolving Cβ2-specific function. Moreover, identifying isoform-specific interaction partners and their interaction domains would most certainly provide valuable insights. This could be achieved by co-precipitation experiments with the alternative Cα and Cβ N-termini in different cell and tissue lysates in which the various PKA C subunit isoforms are known to be highly expressed. It would also be of interest to systematically point mutate amino acids defining the signature residues in Cα1 and Cβ1, respectively, as well as the amino acids responsible for forming structures such as the α-helix in the Cβ2 N-terminus and any other amino acids that may define vital and important functional features of activity and location. The latter may be achieved by applying the CRISPR/Cas9 technique and studies of mutated proteins at both the cellular and animal level [56, 57].

## Materials & methods

Orthologs of human *PRKACA* and *PRKACB* were obtained from the preclustered gene data available in Ensembl [58] and from the NCBI RefSeq and non-redundant database resources [59, 60] employing standard BLAST [61] sequence searching.

In order to obtain transcriptional data for the *PRKACA* and *PRKACB* orthologs in a particular organism, the NCBI Sequence Read Archive (SRA) resource was used [45]. Standard Entrez [59] was employed to search for RNA-Seq studies and data sets deposited in the SRA for each particular organism and tissue, and the raw data sets were searched with the NCBI SRA nucleotide BLAST tool (megablast or blastn [61]). Note that unlike other NCBI sequence databases, the individual raw data sequences in the SRA do not have readily searchable sequence identifiers available through Entrez. All sequences reported in this study either corresponds to an NCBI RefSeq sequence or they were generated by aligning SRA reads. In the latter case, all sequences presented are matching at least 20 SRA reads over their full length (See also Supplementary S1 Methods and S1 Fig).

Multiple sequence alignments were generated with MAFFT [62], and the multiple sequence alignments were edited and viewed with Jalview [63]. PyMOL (W. L. DeLano, The PyMOL Molecular Graphics System, Version 1.8, Schrödinger, LLC) was used for all protein structure illustrations, and protein structural disorder was predicted with DISOPRED3 [52]. Signature sequence logos were generated with WebLogo [64].

## Supporting information

S1 Methods Sequence assembly exemplified through generation of the *Callorhinchus milii* (Australian ghostshark) PKA Cα1 sequence. (PDF)

S1 Table New full length PKA catalytic subunit homologs assembled in present work, nucleotide sequences. (PDF)

S2 Table New full length PKA catalytic subunit homologs assembled in present work, protein sequences. (PDF)

S1 Fig Model of RNA-Seq data analysis. Illustration of how the NCBI SRA reads from RNA-Seq projects were used to assemble the 5’ region (N-terminus of encoded protein sequence, shown under the alignment) of PKA Cα1 of Australian ghostshark (*Callorhinchus milii*). XM_007909379.1 is the NCBI RefSeq identifier for the transcript predicted from the *C*. *milii* genome, while SRR514109, SRR513759, and SRR513760 are Illumina sequencing RNA-Seq raw data sets submitted by the Elephant shark Genome Project, Institute of Molecular and Cell Biology, Singapore, from shark brain, ovary, and liver, respectively. As an illustration of the amount of data available, the sizes of these three datasets are approximately 70, 52, and 110 million sequence reads, respectively. See Supplementary Methods for details.
(PNG)

S2 Fig Multiple sequence alignment of N-termini of eutherian PKA Cα1/Cβ1 homologs. All sequences were obtained by BLAST sequence searching in the NCBI RefSeq databases. The alignment was used to create sequence logos for eutherian PKA Cα1 and Cβ1 N-termini as depicted in Fig 3D.
(PNG)
