# Supplementary material for: Evolution of the cAMP-dependent protein kinase (PKA) catalytic subunit isoforms
Source: PLoS One. 2017 Jul 25;12(7):e0181091. doi: 10.1371/journal.pone.0181091 (PMC5526564; doi:10.1371/journal.pone.0181091)
Supplement: S1 Methods — (PDF) [file pone.0181091.s001.pdf]

S1 Methods. Sequence assembly exemplified through generation of the *Callorhinchus milii* (Australian ghostshark) PKA Cα1 sequence.

The main focus of the current paper was to identify alternative exons located 5' of exon 2 in the genes *PRKACA* and *PRKACB* of vertebrates. Starting with the known sequence of *PRKACA* exon 2 in the ghostshark genome (NCBI RefSeq identifier XM_007909379.1), a segment of approximately 60 nucleotides, beginning at the exon 2 5' end, was used as a query sequence. BLAST sequence searching in the NCBI Sequence Read Archive (SRA) resource was performed in the datasets from transcriptomics projects (RNA-Seq studies) from a range of different tissues. This typically resulted in over a thousand hits with highly similar (mainly identical for the aligned segments) sequences, many of them with 5' flanking sequences, reflecting mRNA with alternative 5' exons. Translated nucleotide sequences were aligned, and revealed the alternative 5' exons in the respective tissues. The resulting variants were used as new query sequences for additional BLAST searches in order to obtain more upstream mRNA sequence, and this protocol was generally performed iteratively until a putative start codon was identified. An alignment of a small subset of SRA sequences acquired through this process is shown in S1 Fig. In general, the alternative 5' exons and their splicing with exon 2 were supported by several hundred SRA reads, while sequence variants found only once or in few copies were not regarded as significant (*i.e.* they are most likely due to sequencing errors, chimeric RNA or similar). The final *C. millii* PKA Cα1 mRNA and protein sequences are shown in S1 and S2 Tables, respectively. This sequence variant is not available in the RefSeq database, most likely due to missing sequence in the *C. millii* reference genome.

Collection and assembly of sequences for other splice variants and species was performed in a similar fashion for all reported sequences that are not readily available in NCBI RefSeq or other public databases. All alternative 5' exons and their splicing with exon 2 were supported by at least 20 SRA reads, in most cases several hundred.
