# Supplementary material for: Evolution of the cAMP-dependent protein kinase (PKA) catalytic subunit isoforms
Source: PLoS One. 2017 Jul 25;12(7):e0181091. doi: 10.1371/journal.pone.0181091 (PMC5526564; doi:10.1371/journal.pone.0181091)
Supplement: S1 Table — (PDF) [file pone.0181091.s002.pdf]

Species	PKA catalytic subunit nucleotide sequence in FASTA format
Australian ghostshark<br>(*Callorhinchus milii*)	>PKACa1_Callorhinchus_milii<br>ATGGGCAACGCCGCCACCGCCAGGAAGGGCGGCGAAGTGGACAGCGTGAAAGAATTCTTAGAGAAAGCCAAA<br>GAGGAGTTCTTACGGAAATGGGAGAGTCCGACACAGAACACGGCGAATCTGGAAGATTTTGAAAGAACAAAA<br>ACCCTGGGCACAGGGTCGTTTGGCCGAGTGATGCTGGTGAAGCACAAAGTCACACAGCAGCATTACGCCATG<br>AAGATCCTCGACAAACAGAAGGTGGTAAAACTGAAGCAGATTGAACACACGCTGAATGAGAAAAGGATTCTT<br>CAGGCTGTCAATTTCCCTTTCCTGGTGAGGCTAGAGTACTCGTTCAAGGATAACTCTAATTTATATATGGTG<br>ATGGAATACGTTTCAGGAGGAGAGATGTTTTCACATTTAAGGAGAATCGGGAGGTTCAGTGAACCTCACGCA<br>CGGTTTTACGCATCACAGATCGTCCTTGCCTTCGAATACCTCCACTCACTCGACCTCGTCTACAGAGACCTG<br>AAGCCTGAAAACATACTGATCGACCAATATGGATACATCCAGGTGACAGATTTTGGCTTTGCCAAGAGAGTA<br>AAGGGCAGAACTTGGACGCTATGTGGGACTCCCGAATACCTGGCTCCTGAGATCATCCTCAGCAAGGGCTAC<br>AACAAAGCTGTGGATTGGTGGGCGTTAGGGGTCCTCATTTACGAAATGGCTGCTGGTTACCCCCCATTTTTT<br>GCAGACCAGCCTATTCAGATTTATGAGAAGATAGTCTCTGGGAAGGTCCGGTTTCCGTCTCATTTCAGCTCA<br>GACCTGAAGGACCTGCTGCGAAACCTGCTGCAGGTTGACCTGACCAAACGGTTCGGGAATCTCAAGAACGGC<br>GTCAACGACATCAAGAACCACAAGTGGTTTGCCATCACAGACTGGATCGCCATCTACCAGAAAAAGGTTGAA<br>CCTCCTTTTATACCAAAGTACAAAGGTCCCGGCGACTCCAGCAACTTTGACGACTATGAAGAAGAGGAGGTC<br>CGAGTGTCTGTCACCAACAAATGTTCCAAGGAATTTCTGGACTTCTAG<br><br>>PKACb1_Callorhinchus_milii<br>ATGGGAAACACTGCGACAGCCAAAAAGGGCAACGAAATTGAGAGCGTGAAAGAGTTCTTAGCCAAAGCCAAA<br>GAAGATTTCTTAAAAAAATGGGAAAGTCCTCCACAGAACACTGCAGGCCTAGATGATTTTGAAAGACAAAAA<br>ACACTTGGAACGGGTTCTTTTGGAAGAGTTATGTTGGTGAAACATAAAGGTACAGAGCAGTACTACGCCATG<br>AAGATATTGGATAAACAGAAAGTTGTCAAACTGAAGCAAATAGAGCATACACTCAATGAGAAAAGAATATTG<br>CAAGCTGTTAATTTCCCATTTCTGGTGAAGTTGGAGTATTCCTTTAAGGACAACTCTAATTTATATATGGTG<br>ATGGAGTACGTTCCAGGTGGTGAAATGTTTTCACATCTTAGGAGAATTGGAAGGTTCAGTGAACCTCATGCA<br>CGATTTTATGCAGCTCAAATAGTCCTGACCTTTGAGTACCTCCATTCACTAGACCTCATCTACAGAGATCTG<br>AAGCCAGAAAACCTTTTAATTGACCAACAAGGATACATCCAGGTCACAGATTTTGGTTTTGCAAAAAAGGTA<br>AGGGGCAGAACTTGGACGCTTTGTGGAACTCCAGAATACTTGGCTCCAGAGATTATTCTCAGCAAGGGCTAC<br>AACAAAGCAGTGGACTGGTGGGCACTGGGGGTTCTAATCTATGAAATGGCAGCTGGATATCCTCCTTTCTTT<br>GCAGACCAGCCTATACAGATCTATGAAAAGATTGTCTCTGGAAAGGTACGGTTCCCTTCTCACTTCAGTTCA<br>GACCTGAAAGACCTGCTGCGAAATTTACTTCAGGTAGATCTGACCAAACGATATGGGAATCTCAAGAACGGT<br>GTGAATGACATTAAGAACCACAAGTGGTTTGCCACTACAGATTGGATTGCCATTTATCAGAGAAAGGTGGAA<br>GCGCCATTCATACCAAAGTGCAGAGGTCCTGGAGATACCAGCAATTTTGATGATTATGAAGAGGAGGACATC<br>CGTGTCTCTTTAACCGAAAAGTGTGCTAAAGAATTTGCTGATTTTTAG
Sea lamprey<br>(*Petromyzon marinus*)	>PKACx1_Homolog1_Petromyzon_marinus<br>ATGGGGAACGCGGCGGCGACAAAGAAAGGGGGCGAGGCGGAGAGCGTGAAAGAGTTCCTGGCGAAGGCGAAG<br>GAAGATTTTATGCGAAAATGGGAAAACCCACAGCAGAACACGTCGTGCCTGGAGGACTTTGAGCGAATAAAG<br>ACGCTTGGCACAGGCTCGTTCGGCCGCGTCATGCTCGTCAAGCACAAGTCCAGCGACCAGTTTTTCGCCATG<br>AAGATCCTCGACAAACAGAAGGTGGTCAAGTTAAAACAAGTGGAACACACGCTCAATGAGAAACGCATCCTG<br>CAGGCCATCAGCTTCCCTTTCCTTGTGCGCCTCGAATACAGCTTCAAGGACAATTCCAACCTCTACATGGTG<br>CTGGAGTACGTGCCCGGTGGAGAGATGTTCTCACATCTGCGCAGGATCGGCCGCTTCAGTGAGCCTCACTCA<br>CGGTTCTACGCGGCACAAATCGTGTTGGCGTTCGAGTACCTGCACTCGTTGGACCTCATCTACCGGGACCTC<br>AAGCCTGAGAACCTCCTTATTGACCAGCAAGGATACATCCAGGTGACAGACTTTGGGTTTGCTAAGCGCGTG<br>AAGGGGCGAACCTGGACTCTGTGTGGAACACCAGAGTATCTGGCTCCCGAGATCATCCTCAGCAAGGGCTAC<br>AACAAAGCGGTTGACTGGTGGGCGCTGGGCGTGCTCATCTATGAGATGGCGGCTGGATACCCGCCATTCTTC<br>GCCGACCAACCCATCCAGATATACGAGAAGATCGTCTCTGGGAAGGTGCGGTTCCCTTCTCACTTCAGCTCG<br>GACCTCAAGGACCTGCTGAGGAACCTGCTGCAAGTGGACCTCACCAAGCGATTCGGAAACCTCAAGAACGGT<br>GTCAACGACATCAAGAACCATAAGTGGTTCTCCACCACCGACTGGATCGCAATTTACCAACGCAAGGTCGAG<br>GCTCCCTTCATTCCCAAGTGCAAGGGCCCAGGCGACGCCAGCAATTTTGACGACTACGAGGAGGAGGAAATC<br>CGCATCTCGTCCACTGAGAAGTGCGCCAAGGAGTTTGCCGACTTCTGA<br><br>>PKACx1_Homolog2_Petromyzon_marinus<br>ATGGGGAACAGCGGAGCCACCACGACGAGGAAAGGCAACGAGGCGGAGAGCGTGAAGGAGTTTTTGGCCAAA<br>GCCAAGGAAGACTTCCTGAAGAAATGGGAAAACCCCCAACAGAACACATCGTGCCTGGACGACTTTGAGCGG<br>ATGAAGACGCTGGGAACGGGCTCCTTCGGGCGAGTCATGCTCGTCAAGCACAAGGCGACGGACCGATACTTC<br>GCCATGAAGATCCTCGACAAGCAGAAGGTGGTGAAGTTAAAACAGGTGGAGCACACGCTCAACGAGAAGCGG<br>ATCCTCCAGGCCATTAGCTTCCCCTTCCTCGTCAGCCTCGAGTACAGCTACAAGGACAACTCTAACCTGTAC<br>ATGGTGCTGGAGTACGTGCCCGGGGGAGAGATGTTCTCCCACCTGCGGAGGATCGGACGCTTCAGCGAGCCC<br>CACTCGCGGTTCTACGCGGCTCAGATCGTGCTGGCGTTCGAGTACCTGCACTCACTCGACCTCATCTACCGA<br>GACCTCAAGCCAGAGAACCTTCTCATCGACCAGCAGGGGTACATCCAGGTCACCGACTTCGGCTTTGCCAAG<br>CGCGTCAAGGGGAGGACCTGGACCCTGTGCGGACCCCGGAGTACCTGGCGCCGGAGATAATCCTCAGTAAA<br>GGCTACAACAAAGCCGTGGACTGGTGGGCGCTGGGCGTGCTGGTGTACGAGATGGCGGCGGGGTACCCCCCC<br>TTCTTCGCCGACCAGCCCATTCAGATCTACGAGAAAATCGTCTCCGGGAAGGTGAGATTCCCCTCGCACTTC<br>AGCTCAGACCTGAAGGACGTGCTGAGGAACCTGCTCCAGGTGGACCTCACCAAGCGCTTCGGAAACCTCAAG<br>AACGGCGTCAACGACATCAAGGGGCACAAGTGGTTCTCCACCACCGACTGGATCGCCATCTACCAGCGCAAG<br>GTGGAGGCTCCCTTCATCCCCAAGTGCAAGGGCCCCGGCGACGCCAGCAACTTCGACGACTACGAGGAGGAG<br>GAGATCCGCATCTCCTCCACCGAGAAGTGCGCAAAAGAGTTTGCCGACTTCTAA

Golden eagle<br>(*Aquila chrysaetos*)	>PKACa1_Aquila_chrysaetos<br>ATGGGCAACGCCGCCGCCGCCAAGAAGGGCGGGGAGCTGGAGAGCGTGAAGGAGTTTTTGGCCCGGGCAAAG<br>GAGGATTTTCTCAAAAAATGGGAGAACCCAGCCCAGAACACAGCCAGCCTGGAGCAGTTCGAGCGCATCCGC<br>ACGCTGGGGACGGGCTCCTTCGGGCGCGTCATGCTGGTGCGGCACAGGGACACGGGCCACCACTACGCCATG<br>AAGATTCTCGATAAGCAGAAGGTGGTGAAGCTGAAGCAGATCGAGCACACGCTGAATGAGAAGCGGATCCTG<br>CAGGCTGTCACCTTCCCCTTCCTCGTCCGCCTCGAGTACTCCTTCAAGGACAACTCCAACCTGTACATGGTG<br>ATGGAGTACATCCCGGGGGGGGAGATGTTCTCCCACCTCCGCCGCATCGGCCGCTTCAGTGAGCCCCACGCC<br>CGATTCTACGCGGCCCAAATCGTGCTGACCTTTGAGTACCTGCACGCGCTTGACCTCATCTACCGCGACCTG<br>AAACCTGAAAACCTGCTCATCGACCAGCAGGGCTACATCGAGGTGACAGATTTCGGCTTCGCCAAGCGGGTG<br>AAGGGCCGGACCTGGACGCTGTGCGGCACCCCCGAGTACCTGGCACCCGAAATCATCCTCAGCAAGGGGTAC<br>AACAAGGCTGTGGACTGGTGGGCCCTGGGGGGCCTTATCTACGAGATGGCGGCCGGGTACCCCCCCTTCTTC<br>GCCGACCAGCCCATCCAGATCTACGAGAAGATCGTCTCGGGGAAGGTCCGGTTCCCCTCGCACTTCAGCTCG<br>GACCTGAAGGACCTCCTGCGCAACCTGCTCCAGGTCGACCTGACCAAGCGCTTCGGGAACCTCCGTAACGGC<br>GTCGCCGACATTAAAGGCCACAAATGGTTCTCCACCACCGACTGGATCGCCATCTACCAGCGCAAGGTGGAA<br>GCCCCCTTCGTGCCCAAGTGCAAAGGCCCCGGGGACACCAGTAACTTCGACGAGTACGAGGAGGAAGAGATC<br>CGCGTCTCCCTCACCGACCGCTGCGCCAAGGAGTTTGCCGACTTCTAG
