# Supplementary material for: Evolution of the cAMP-dependent protein kinase (PKA) catalytic subunit isoforms
Source: PLoS One. 2017 Jul 25;12(7):e0181091. doi: 10.1371/journal.pone.0181091 (PMC5526564; doi:10.1371/journal.pone.0181091)
Supplement: S2 Table — (PDF) [file pone.0181091.s003.pdf]

Species	PKA catalytic subunit protein sequence in FASTA format
Australian ghostshark (*Callorhinchus milii*)	>PKACa1_Callorhinchus_milii<br>MGNAATARKGGEVDSVKEFLEKAKEEFLRKWESPTQNTANLEDFERTKTLGTGSFGRVMLVKHKVTQQHYAM<br>KILDKQKVVKLKQIEHTLNEKRILQAVNFPFLVRLEYSFKDNSNLYMVMEYVSGGEMFSHLRRIGRFSEPHA<br>RFYASQIVLAFEYLHSLDLVYRDLKPENILIDQYGYIQVTDFGFAKRVKGRTWTLCGTPEYLAPEIILSKGY<br>NKAVDWWALGVLIYEMAAGYPPFFADQPIQIYEKIVSGKVRFPSHFSSDLKDLLRNLLQVDLTKRFGNLKNG<br>VNDIKNHKWFAITDWIAIYQKKVEPPFIPKYKGPGDSSNFDDYEEEEVRVSVTNKCSKEFLDF<br><br>>PKACb1_Callorhinchus_milii<br>MGNTATAKKGNEIESVKEFLAKAKEDFLKKWESPPQNTAGLDDFERQKTLGTGSFGRVMLVKHKGTEQYYAM<br>KILDKQKVVKLKQIEHTLNEKRILQAVNFPFLVKLEYSFKDNSNLYMVMEYVPGGEMFSHLRRIGRFSEPHA<br>RFYAAQIVLTFEYLHSLDLIYRDLKPENLLIDQQGYIQVTDFGFAKKVRGRTWTLCGTPEYLAPEIILSKGY<br>NKAVDWWALGVLIYEMAAGYPPFFADQPIQIYEKIVSGKVRFPSHFSSDLKDLLRNLLQVDLTKRYGNLKNG<br>VNDIKNHKWFATTDWIAIYQRKVEAPFIPKCRGPGDTSNFDDYEEEDIRVSLTEKCAKEFADF
Sea lamprey (*Petromyzon marinus*)	>PKACx1_Homolog1_Petromyzon_marinus<br>MGNAAATKKGGEAESVKEFLAKAKEDFMRKWENPQQNTSCLEDFERIKTLGTGSFGRVMLVKHKSSDQFFAM<br>KILDKQKVVKLKQVEHTLNEKRILQAISFPFLVRLEYSFKDNSNLYMVLEYVPGGEMFSHLRRIGRFSEPHS<br>RFYAAQIVLAFEYLHSLDLIYRDLKPENLLIDQQGYIQVTDFGFAKRVKGRTWTLCGTPEYLAPEIILSKGY<br>NKAVDWWALGVLIYEMAAGYPPFFADQPIQIYEKIVSGKVRFPSHFSSDLKDLLRNLLQVDLTKRFGNLKNG<br>VNDIKNHKWFSTTDWIAIYQRKVEAPFIPKCKGPGDASNFDDYEEEEIRISSTEKCAKEFADF<br><br>>PKACx1_Homolog2_Petromyzon_marinus<br>MGNSGATTTRKGNEAESVKEFLAKAKEDFLKKWENPQQNTSCLDDFERMKTLGTGSFGRVMLVKHKATDRYF<br>AMKILDKQKVVKLKQVEHTLNEKRILQAISFPFLVSLEYSYKDNSNLYMVLEYVPGGEMFSHLRRIGRFSEP<br>HSRFYAAQIVLAFEYLHSLDLIYRDLKPENLLIDQQGYIQVTDFGFAKRVKGRTWTLCGTPEYLAPEIILSK<br>GYNKAVDWWALGVLVYEMAAGYPPFFADQPIQIYEKIVSGKVRFPSHFSSDLKDVLRNLLQVDLTKRFGNLK<br>NGVNDIKGHKWFSTTDWIAIYQRKVEAPFIPKCKGPGDASNFDDYEEEEIRISSTEKCAKEFADF
Golden eagle (*Aquila chrysaetos*)	>PKACa1_Aquila_chrysaetos<br>MGNAAAAKKGGELESVKEFLARAKEDFLKKWENPAQNTASLEQFERIRTLGTGSFGRVMLVRHRDTGHHYAM<br>KILDKQKVVKLKQIEHTLNEKRILQAVTFPFLVRLEYSFKDNSNLYMVMEYIPGGEMFSHLRRIGRFSEPHA<br>RFYAAQIVLTFEYLHALDLIYRDLKPENLLIDQQGYIEVTDFGFAKRVKGRTWTLCGTPEYLAPEIILSKGY<br>NKAVDWWALGGLIYEMAAGYPPFFADQPIQIYEKIVSGKVRFPSHFSSDLKDLLRNLLQVDLTKRFGNLRNG<br>VADIKGHKWFSTTDWIAIYQRKVEAPFVPKCKGPGDTSNFDEYEEEEIRVSLTDRCAKEFADF
