# Supplementary figures and images for: Evolution of the cAMP-dependent protein kinase (PKA) catalytic subunit isoforms
Source: PLoS One. 2017 Jul 25;12(7):e0181091. doi: 10.1371/journal.pone.0181091 (PMC5526564; doi:10.1371/journal.pone.0181091)

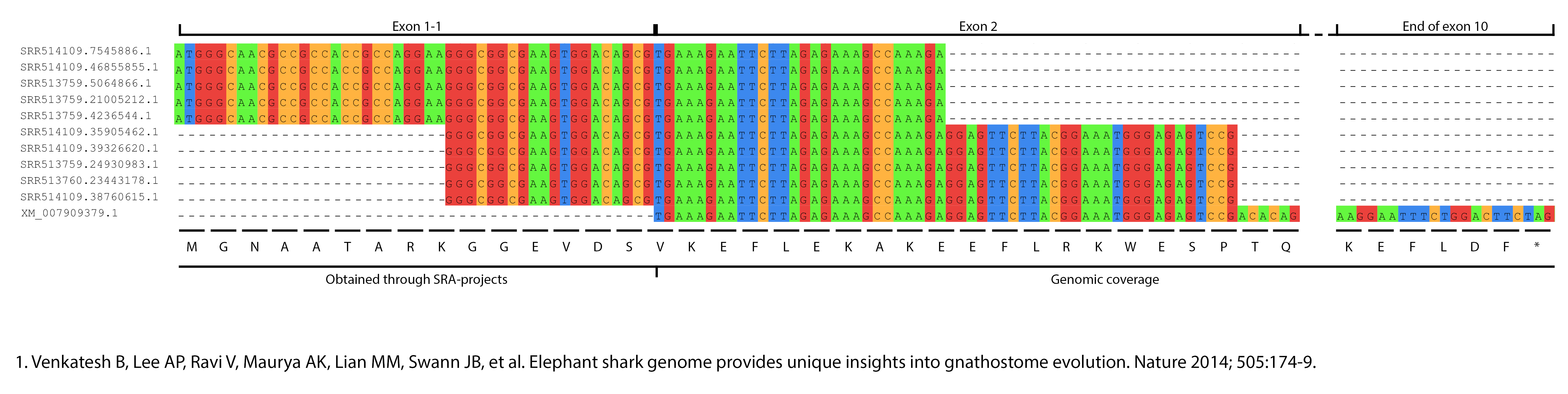

Supplement: S1 Fig — Illustration of how the NCBI SRA reads from RNA-Seq projects were used to assemble the 5’ region (N-terminus of encoded protein sequence, shown under the alignment) of PKA Cα1 of Australian ghostshark (Callorhinchus milii). XM_007909379.1 is the NCBI RefSeq identifier for the transcript predicted from the C. milii genome, while SRR514109, SRR513759, and SRR513760 are Illumina sequencing RNA-Seq raw data sets submitted by the Elephant shark Genome Project, Institute of Molecular and Cell Biology, Singapore, from shark brain, ovary, and liver, respectively. As an illustration of the amount of data available, the sizes of these three datasets are approximately 70, 52, and 110 million sequence reads, respectively. See Supplementary Methods for details. (PNG) [file pone.0181091.s004.png]

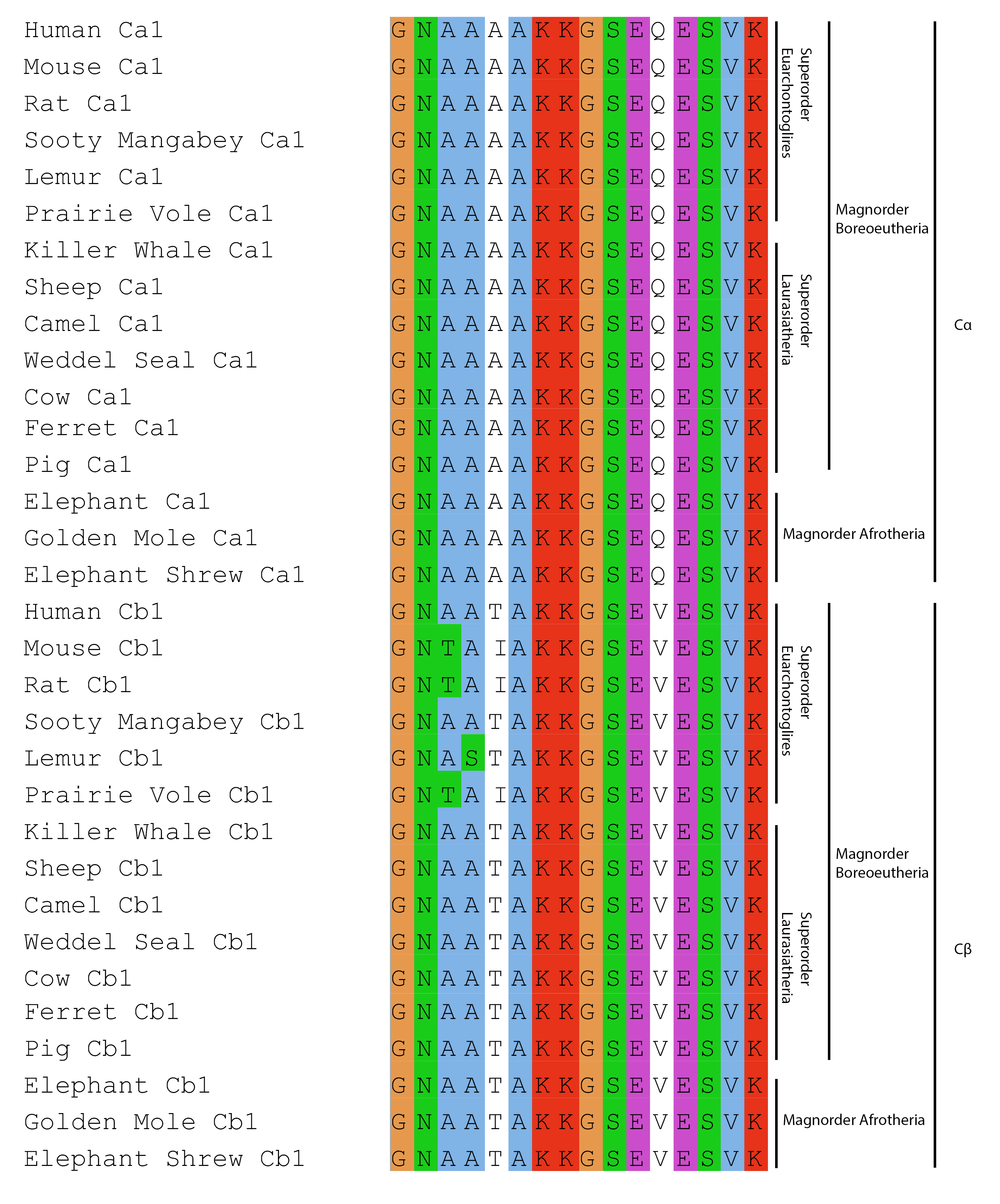

Supplement: S2 Fig — All sequences were obtained by BLAST sequence searching in the NCBI RefSeq databases. The alignment was used to create sequence logos for eutherian PKA Cα1 and Cβ1 N-termini as depicted in Fig 3D. (PNG) [file pone.0181091.s005.png]
